# Bone Regeneration in Rat Cranium Critical-Size Defects Induced by Cementum Protein 1 (CEMP1)

**DOI:** 10.1371/journal.pone.0078807

**Published:** 2013-11-12

**Authors:** Janeth Serrano, Enrique Romo, Mercedes Bermúdez, A. Sampath Narayanan, Margarita Zeichner-David, Leticia Santos, Higinio Arzate

**Affiliations:** 1 Laboratorio de Biología Periodontal, Facultad de Odontología, Universidad Nacional Autónoma de México, México; 2 School of Medicine, Department of Pathology, University of Washington, Seattle, Washington, United States of America; 3 Ostrow School of Dentistry, University of Southern California, Los Angeles, California, United States of America; 4 Instituto Nacional de Investigaciones Nucleares, México; Faculdade de Medicina Dentária, Universidade do Porto, Portugal

## Abstract

Gene therapy approaches to bone and periodontal tissue engineering are being widely explored. While localized delivery of osteogenic factors like BMPs is attractive for promotion of bone regeneration; method of delivery, dosage and side effects could limit this approach. A novel protein, Cementum Protein 1 (CEMP1), has recently been shown to promote regeneration of periodontal tissues. In order to address the possibility that CEMP1 can be used to regenerate other types of bone, experiments were designed to test the effect of *hr*CEMP1 in the repair/regeneration of a rat calvaria critical-size defect. Histological and microcomputed tomography (µCT) analyses of the calvaria defect sites treated with CEMP1 showed that after 16 weeks, *hr*CEMP1 is able to induce 97% regeneration of the defect. Furthermore, the density and characteristics of the new mineralized tissues were normal for bone. This study demonstrates that *hr*CEMP1 stimulates bone formation and regeneration and has therapeutic potential for the treatment of bone defects and regeneration of mineralized tissues.

## Introduction

Large scale bony defects in the cranial skeleton may result of congenital defects, acquired injuries, neurosurgical procedures or infection. Since successful spontaneous calvaria re-ossification rarely occurs, even in young infants [1], the repair of large calvaria bony defects remains a clinical challenge. Bone healing requires coordinated interactions between cells, osteoinductive growth factors, osteoconductive matrix and vascular supply [2], [3]. Regulation of bone metabolism is mediated by both systemic and local factors [4], of which bone morphogenetic proteins (BMPs) appear to be key regulators involved in the formation of new bone, both embryological and in the repair of fractures [5]. It is well known that BMPs, regulate bone formation and promote fracture healing, in part by stimulating the differentiation of non-committed precursor cells into osteoblasts. Studies involving the use of exogenously administered recombinant BMP-2 and BMP-7 have shown that although they are potent inducers of bone healing in lower animals, they might not suffice to regenerate massive bone defects in a compromised host environment [6]–[12]. Nevertheless, gene therapy is a promising approach that can be used to deliver recombinant proteins in *in vivo* models [13]–[15]. Currently available recombinant growth factors approved for clinical use by the Food and Drug Administration (FDA) include human recombinant BMP-2 and BMP-7 [13].

Cementum is a mineralized connective tissue that covers the root surfaces of the teeth, and possesses unique characteristics since it is avascular, does not have innervations and lymphatic drainage and unlike bone, does not suffer physiological remodeling. It provides the interface through which the root surface is anchored to the bone by means of the collagen Sharpey’s fibers of the periodontal ligament. The cementum matrix consists of collagen types I and III [16], fibronectin, osteopontin (OPN), bone sialoprotein (BSP), osteocalcin (OCN), vitronectin (VN) and growth factors such as TGF-β and BMP-2 [17], [18], none of which is cementum-specific. A novel cementum-derived molecule known as CEMP1 (cementum protein 1; GenBank Accession: NM_001048212; NM_001048216; GI: 313677962; HGNC: ID 32553), has been isolated, characterized and shown that it is specifically expressed by cementoblasts, some periodontal ligament (PDL) cell populations and mesenchymal stem cells located paravascularly in the PDL [17], [19]. The high levels of CEMP1 expression in the cementoblastic cell layer and PDL cell populations suggest that CEMP1 might play a role as a local regulator in the differentiation of PDL cell populations [19]. This was further supported by studies showing that transfection of CEMP1 mRNA into human gingival fibroblasts, which do not produce a mineralized extracellular matrix (ECM), changed their phenotype towards a cementoblastic/osteoblastic phenotype. These cells expressed cementum/bone-associated proteins and produced a mineralized ECM [18]. CEMP1 possesses strong affinity for hydroxyapatite and participates in the mineralization process *in vitro.* The purpose of this investigation was to determine if *hr*CEMP promotes bone regeneration in critical-size defects in rat calvaria *in vivo*.

## Materials and Methods

### Ethics Statement

All animal procedures were approved by the Institutional Research, Ethical and Animal Care and Use Committee (Facultad de Odontología, Universidad Nacional Autónoma de México). The animals were euthanized with carbon dioxide gas.

### hrCEMP1 Expression and Purification

Production of human recombinant CEMP1 has been previously described in detail elsewhere [17]. Briefly, recombinant clones were constructed with a pENTR/SD/D-TOPO vector (Invitrogen, Carlsbad, CA, USA) for directional cloning of a blunt-end PCR CEMP1 product. The plasmid was introduced into BL21 (DE3) expression host E. coli strain (Invitrogen, Carlsbad, CA, USA). Human recombinant CEMP1 (*hr*CEMP1) protein was purified by Ni^2+^ affinity chromatography HiTrap Chelating HP column (Invitrogen, Carlsbad, CA, USA, Invitrogen). Protein purity was determined by SDS-PAGE and Western blotting.

### hrCEMP1 Induced Apatite Crystal Formation in a Cell-free System

To determine if *hr*CEMP1 promotes apatite formation, a capillary counter-diffusion system was used [20]–[22]. Briefly, 1% (w/v) agarose gel containing 20 µg/mL of *hr*CEMP1 was poured into capillaries (0.5 mm diameter and 30 mm long). The ends of the capillaries were injected with 100 mM CaCl_2_ and 100 mM NaH_2_PO_4_. All experiments were carried out at 37°C. After 7 days, the crystals were recovered by dissolving the gel into hot milli Q water and air-dried.

### Energy-dispersive X-ray Micro-analysis (EDX)

The composition of crystals formed by induction of *hr*CEMP1 into the capillaries was analyzed using a Jeol 5600 scanning electron microscope fitted with an energy dispersive X-ray microanalysis microprobe. All analyses were performed at 20 kV for 300 s [23]. Crystals were analyzed in low vacuum and the calcium/phosphate (Ca/P) ratio was calculated from the intensity of the peaks present in the EDX pattern. After determining the composition of the crystals, they were covered with a thin gold film, 100 nm thick, to avoid electron disturbances that could interfere with the SEM images.

### Atomic Force Microscopy

Atomic force microscopy (AFM) was used to determine the morphology and homogeneity of the mineralized structures. Histological sections were deparaffinized in xylene and ethanol and air dried. The examination was performed with an AFM (Park Scientific Instruments, Sunnyvale, CA, USA), with an AutoProbe in contact mode with a constant applied force (10 nN) at 1 Hz scan rate in dry samples [24]. The probe was positioned in valleys to allow for the examination of areas that were not affected by the sectioning process of the specimen.

### In vitro Release of hrCEMP1 from Gelatin Matrix Scaffold

Gelatin matrix was cut into disks using a standard 9 mm biopsy punch. Loading was accomplished by application of 25 µg/disc of *hr*CEMP1 to each gelatin matrix disk, left to settle for 1 hr and dried in a lyophilizer, followed by incubation at 37°C for, 1, 3, 24, 6, 24 48, 72, 96 and 168 h. The cumulative release was calculated by summing the amount of *hr*CEMP1 released over the various time intervals. The percentage of cumulative *hr*CEMP1 released is reported as the ratio of the cumulative release of *hr*CEMP1 (in µg) at any point in time to the initial amount of *hr*CEMP1 (in µg) that had been loaded.

### Surgical Procedures

Male Wistar rats aged 7–8 weeks and weighting 250–300 grams were obtained from the Universidad Nacional Autónoma de México, Facultad de Medicina Vivarium, and acclimatized at the Animal Research Facility for 7–14 days before the start of the experiment. The animals were housed in purpose-designed rooms, at a temperature 18–22°C and relative humidity 30–70% with a 12/12 h light/dark cycle. The animals had *ad libitum* access to water and standard laboratory diet. Eight rats were used for each condition. Animals were anesthetized by intraperitoneal injection of 80 mg/kg ketamine and 10 mg/kg xylazine. The surgical site on the dorsal surface of the cranium was prepared by shaving and cleaning with disinfectant. A 3-cm midline incision was made over the calvaria, the skin held open with retractors, the periosteum was pushed to the side bilaterally and care was taken to ensure that the periosteum was completely cleared from the surface of the cranial bone by scraping. A 9-mm craniotomy critical size defect was created with a trephine attached to an electrical drill. Copious saline irrigation was applied during the procedure and the drilling was stopped when the defect area felt loose when probed. The calvarial disk was then removed by severing the remaining connections with a blunt surgical probe. Extreme care was taken to avoid damage to the dura mater, and occasional bleeding was stopped by temporary application of small pieces of gelatin matrix [25]. For implantation, gelatin matrix sponges were cut to the size of the defect (9-mm diameter), and 25 µg of *hr*CEMP1 was pipetted slowly on top of the gelatin matrix in a volume of 200 µl and left to settle for 1 hr and dried in a lyophilizer. Empty gelatin matrix was used as a control and the scaffolds were then inserted into the defect. The surgical site in all conditions was covered with a thin gelatin membrane. The skin was closed using 4-0 silk sutures. After 16 weeks, animals were euthanized using carbon dioxide gas, and the implants embedded in the surrounding native bone were retrieved.

### Histological Analysis

After X-ray imaging and densitometry, calvariae were fixed overnight in 10% formaldehyde as described elsewhere [24]. Following fixation, the specimens were decalcified in 0.5% formaldehyde containing 10% EDTA, pH 7.4, at 4°C for 5 wks., dehydrated in a graded alcohol series, embedded in paraffin, and 5 µm thick sections were prepared and stained with H&E (hematoxylin and eosin). Sections were perpendicular to the sagittal suture in order to produce a plane of analysis through the center of the defect. Three central sections/defect were used for histometric/histological analysis. Additionally, three central sections were stained with Masson’s trichrome for cross reference.

### Analysis of Bone Regeneration

The decalcified tissue sections were stained with H&E for histological examination and quantification of bone regeneration. Bridging of the defects with tissue was examined for all conditions via light microscopic analysis. The mineralized tissue area normalized to total tissue area were computed using Image Pro Plus Software. To confirm that the 9 mm defect was critical-sized, we also created a defect without implanting a scaffold, and examined this condition after 16 weeks. Hematoxylin–eosin stained specimens were used to measure bone viability in the experimental specimens and host bone, the number of osteocyte-occupied lacunae versus empty lacunae and osteoblasts were counted during these analyses in five randomly selected fields at 400× using a 40× lens and 10× eyepiece (Axioskop 2, Carl Zeiss instruments, Germany). In addition, one specimen from each condition after 16 weeks was imaged at a resolution of 9 µm using a three-dimensional µCT system, and reconstructed at a resolution of 18 µm in order to visualize the volume of new bone formation, and qualitatively examine the microarchitecture of the regenerated bone tissue [26].

### Immunofluorescence Analysis

Calvaria sections were incubated with rabbit polyclonal primary antibodies against human OCN and human BSP (Santa Cruz Biotechnologies, Santa Cruz, CA, USA) at a 1∶300 dilution in PBS containing 2 mg/mL BSA. The secondary antibodies for immunostaining, Alexa-Fluor 488 conjugated goat anti-rabbit (dilution 1∶25) were purchased from Invitrogen (Carlsbad, CA, USA). Immunofluorescence was performed as described elsewhere [27]. Negative controls were achieved by omitting the primary antibody or by incubating with normal rabbit serum.

### Statistical Analysis

Eight animals were used for control and experimental groups and values are expressed as mean ± S. E. Multiple comparisons between treatment groups were made with the Neuman-Keuls post-hoc test with a two-way analysis of variance (ANOVA). P<0.05 was considered statistically significant. Statistical analyses were performed with Sigma Stat V 3.1 software (Jandel Scientific Ashburn, VA).

## Results

### hrCEMP1 Induces Formation of Octacalcium Phosphate Crystals in vitro

**Figure 1:**
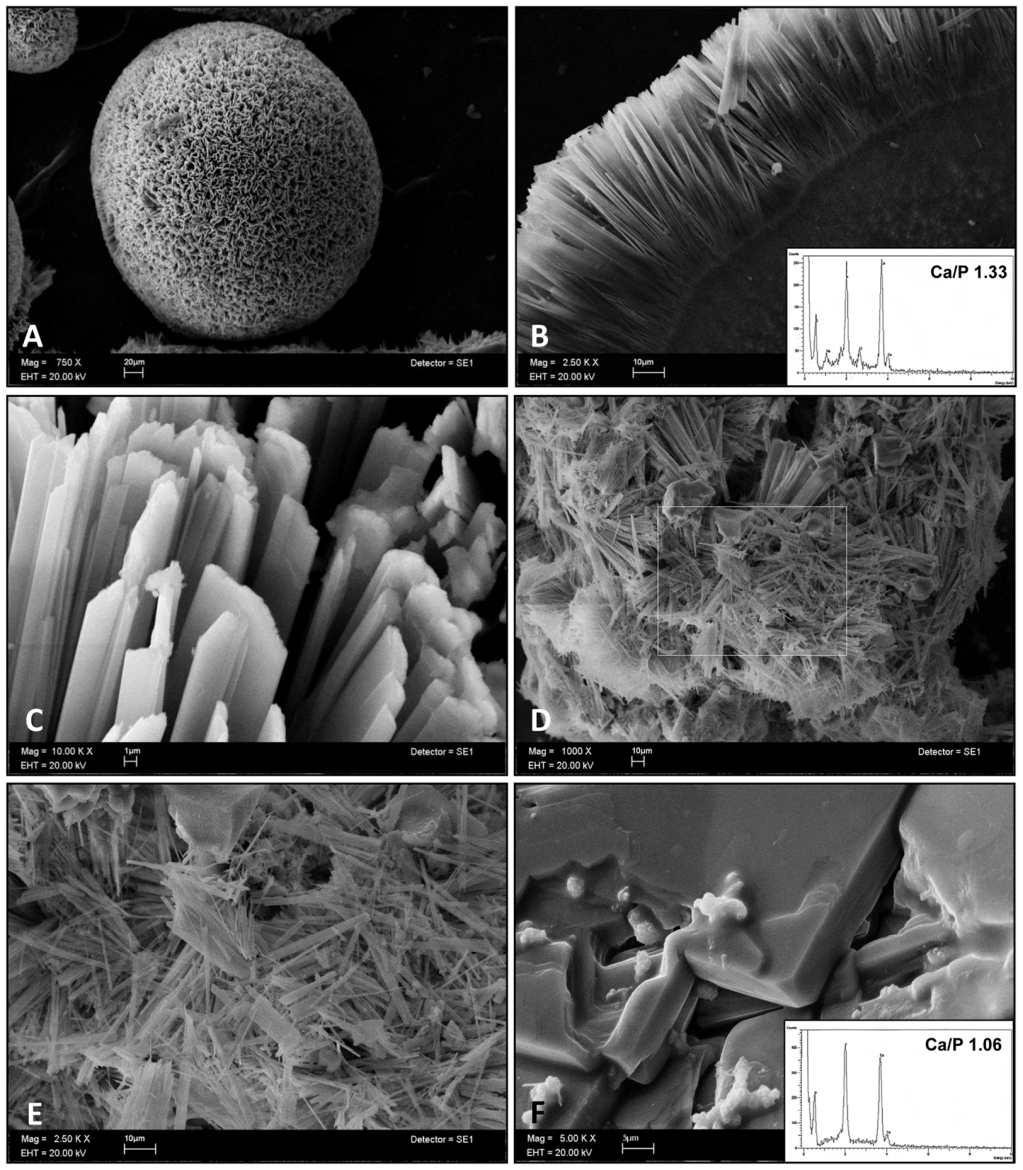
Crystal morphological characterization. (A) SEM image of spheres-like structures formed by *hr*CEMP1. (B) Crystals irradiate from a mineralized core. EDS of apatite crystals showing a Ca/P ratio of 1.33, as expected for OCP (insert in B). SEM image of organized flattened-like crystals nucleated in the presence of 20 µg of *hr*CEMP1(C). Microribbon-like crystals nucleated in the presence of *hr*CEMP (D and E). Plate-like crystals induced by control protein (BSA) (F) and the EDS revealed a Ca/P ratio of 1.06 (Insert in F).

SEM analysis of crystals formed *in vitro* indicates that the effect of *hr*CEMP1 on the growth of octacalcium phosphate (OCP) crystals was unique (Fig. 1). In the presence of *hr*CEMP1, the majority of the crystals organized into microspheres with a mineralized core. The size distribution of the microspheres showed a range between 155 µm up to 249 µm with an average of 194 µm (Fig. 1A and Fig. S1). The crystals irradiating from a nucleus showed needle-like morphology (Fig. 1B). When observed in detail, the OCP crystals showed a prismatic or basaltic morphology as a result of *hr*CEMP1 induction (Fig. 1C). Some OCP crystals grown in the presence of *hr*CEMP1 showed a characteristic ribbon-like morphology (Fig. 1D and E). The crystals showed a hexagonal feature and the broadness average was of 1.32 µm. The average thickness of the OCP crystals was of 0.89 µm (Fig. S1). In contrast, the absence of *hr*CEMP1 resulted in crystals growing in a raft-plaque-like shape (Fig. 1F). Elemental analysis performed with EDX showed that the Ca/P ratio to be 1.06 for control crystals (see spectra insert in 1F), whereas, experimental conditions using 20 µg/mL of *hr*CEMP1, indicate that the crystals are OCP, (Ca_8_H_2_(PO_4_)65H_2_O) since the Ca/P ratio was of 1.33 according to ICDD file: PDF#26-1056 (see spectra insert in 1B).

### CEMP1 Release

Human recombinant CEMP1 was rapidly released from gelatin matrix. Interpolation of the release profile indicates that the time required for 55 and 87% was at 1 and 24 hr respectively. After 24 hr the rate of *hr*CEMP1 release from gelatin matrix remained constant. The amount of CEMP1 was related to the concentration of the hrCEMP1. At 1 hr 13.6 µg (54.8%) were released from the gelatin matrix and at 24 h the maximum amount of released protein was of 21.7 µg (86.8%). After this point the released protein average up to 7 days was of 22.1 µg (88.4%).

### Healing of Calvarial Defect After Implantation of Gelatin Matrix Containing hrCEMP1

**Figure 2:**
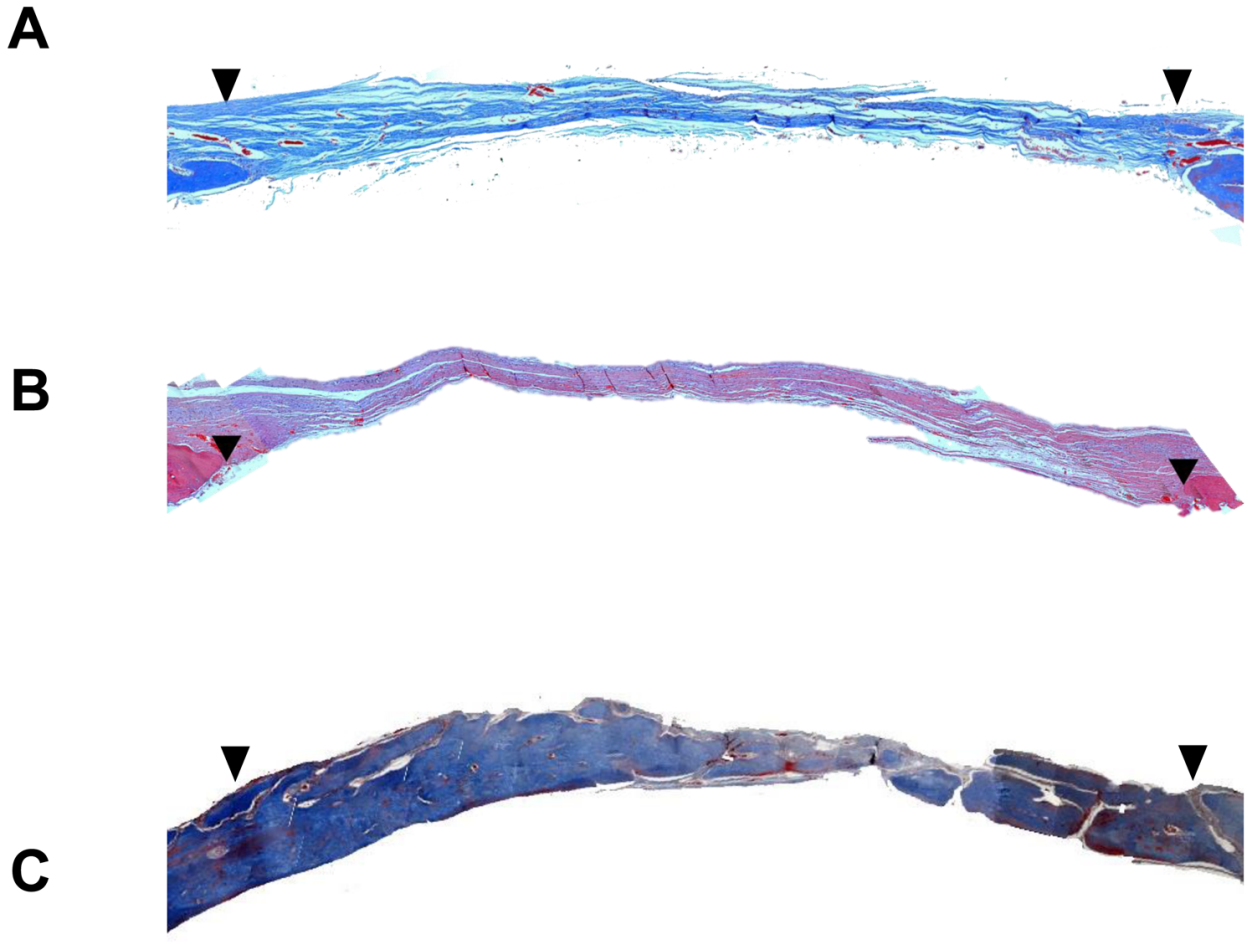
Histological sections stained with H&E show the rat calvaria critical-size defect after 16 weeks. (A) Empty defect; margins of the defect are connected by a thin dense connective fibrous tissue. (B) Gelatin matrix scaffold treatment shows that the defect is occupied by dense fibrous connective tissue. (C) Gelatin matrix scaffold containing *hr*CEMP1 shows that the defect is almost filled with bone-like tissue. All photomicrographs were taken at 100x. Arrowheads show the border of the calvarial defect.

**Figure 3:**
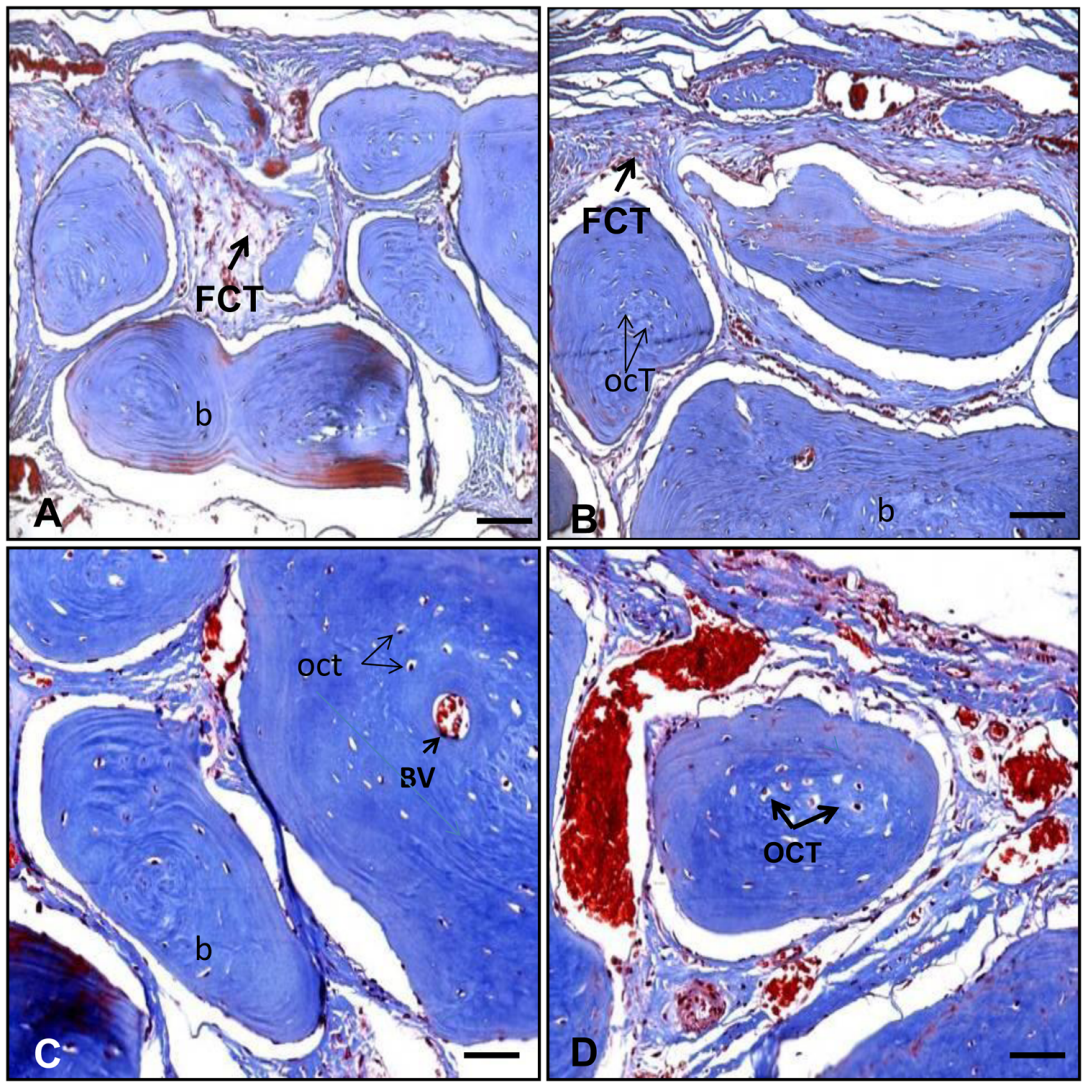
Histological sections of the rat calvaria critical-size defect treated with *hr*CEMP1 and stained with Masson’s trichrome. The pictures show bonny tissue islands limited by dense fibrous connective tissue (FCT) (A and B). The bone-like tissue formed showed lamellar features with the presence of, blood vessels (BV) and embedded osteocytes (OCT) from (C and D). All microphotographs were taken at 400x.

Gelatin matrix scaffolds incorporating *hr*CEMP1 and blank gelatin matrix scaffolds were implanted into rat calvaria critical-sized defects to examine orthotropic bone regeneration. To confirm that the 9 mm defect was critical-sized, and thus would not heal without intervention, a control defect without scaffold was analyzed after 16 weeks post-surgery. Histological analysis revealed that this defect displayed growth of a thin connective fibrous tissue layer but no evidence of bone formation was detected (Figure 2A). Defects implanted with gelatin matrix scaffolds were bridged with a thicker layer of fibrous connective tissues (Figure 2B). In contrast, experimental scaffolds of gelatin matrix containing *hr*CEMP1 showed almost complete filling of the cranial defect with a mineralized type of tissue resembling the calvarial tissue present at the edges of the defect (Fig. 2C). Further analysis at higher magnification using sections stained with Masson’s trichrome for bone showed large amounts of bony nodules, both in number and size, which led to the formation of thin strips of bony tissue. These bony tissue islands were surrounded by dense connective tissue (Fig. 3A–B). The newly formed bone-like tissue shows clearly morphological characteristics of normal bone with osteocytes embedded in their lacuna and osteoblasts lining the outer edge of the bone tissue (Fig. S2). In certain areas, bone formation consisted of both evolving immature woven bone, and mature bone, as characterized by a lamellar structure with interconnected bony trabeculae, and the presence of blood vessels inside bone-like tissue was evident (Fig. 3C–D). There was no evidence of inflammatory response in the experimental or control conditions.

### Molecular Analysis of the Filled Defect

**Figure 4:**
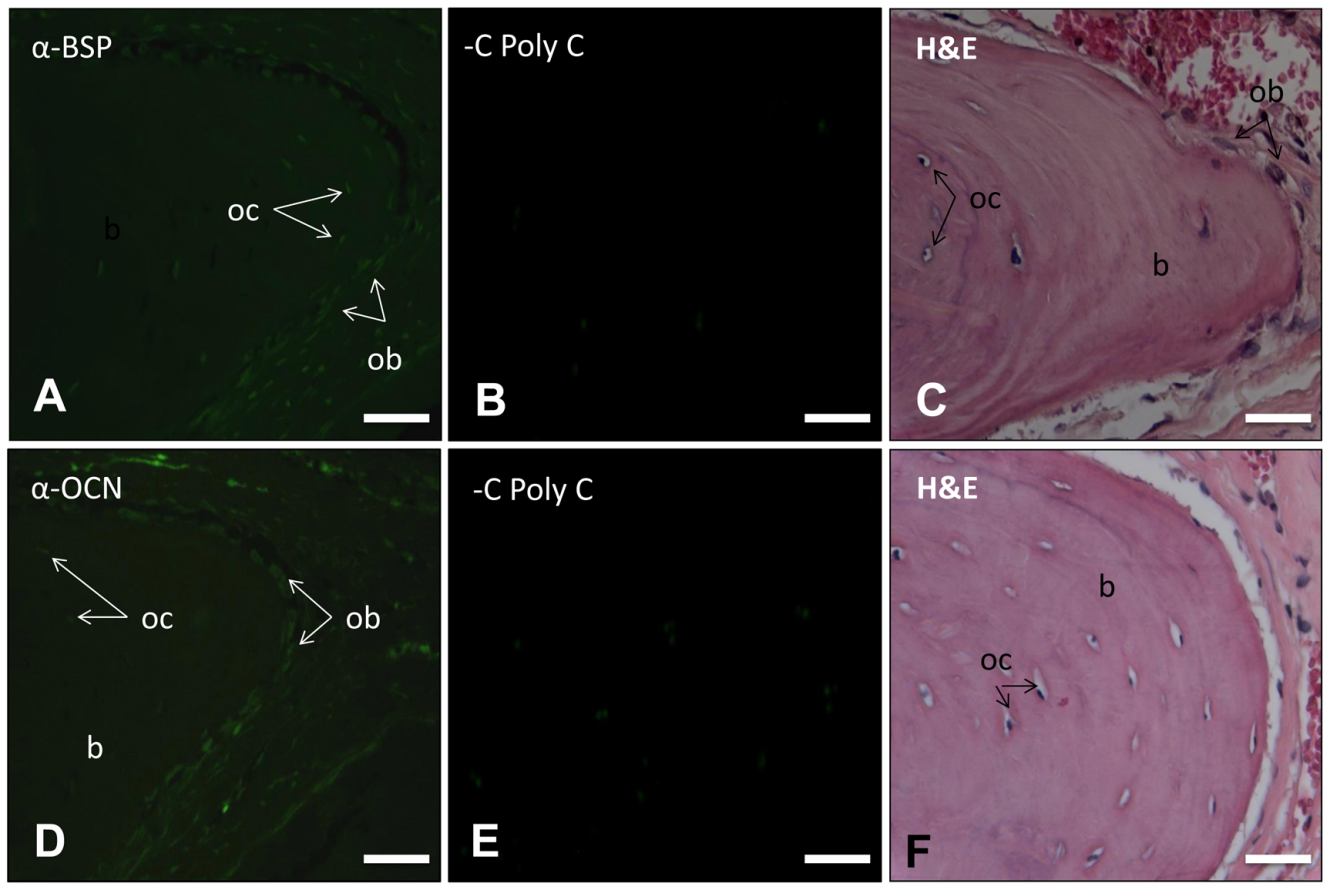
The tissue formed in the rat calvaria defect in the presence of gelatin matrix containing *hr*CEMP1 was examined for the expression of bone-related proteins. The presence of BSP (A) and OCN (D) can be seen in the newly formed tissue filling the defect. The presence of these proteins was confined to the cells surrounding the mineralized matrix (osteoblasts, ob) on the osteoid front and in osteocytes (oct). Pictures C and F are H & E stained for morphological orientation. B and E represent negative controls. All microphotographs were taken at 400x.

In order to assess the molecular identity of the filled defect, the presence and expression of bone-related molecules was analyzed by immunohistochemistry using fluorescent detection. BSP was present in cells surrounding mineralized rounded structures and osteoblasts facing the mineralized front of bony tissue as well as osteoid tissue and osteocytes immersed into the mineralized matrix were positive (Fig. 4A). OCN was detected in a similar distribution as BSP (Fig. 4D. Pre-immune rabbit serum negative controls were negative. (Fig. 4B and E respectively).

### X-ray Micro-analysis (EDX) and AFM

**Figure 5:**
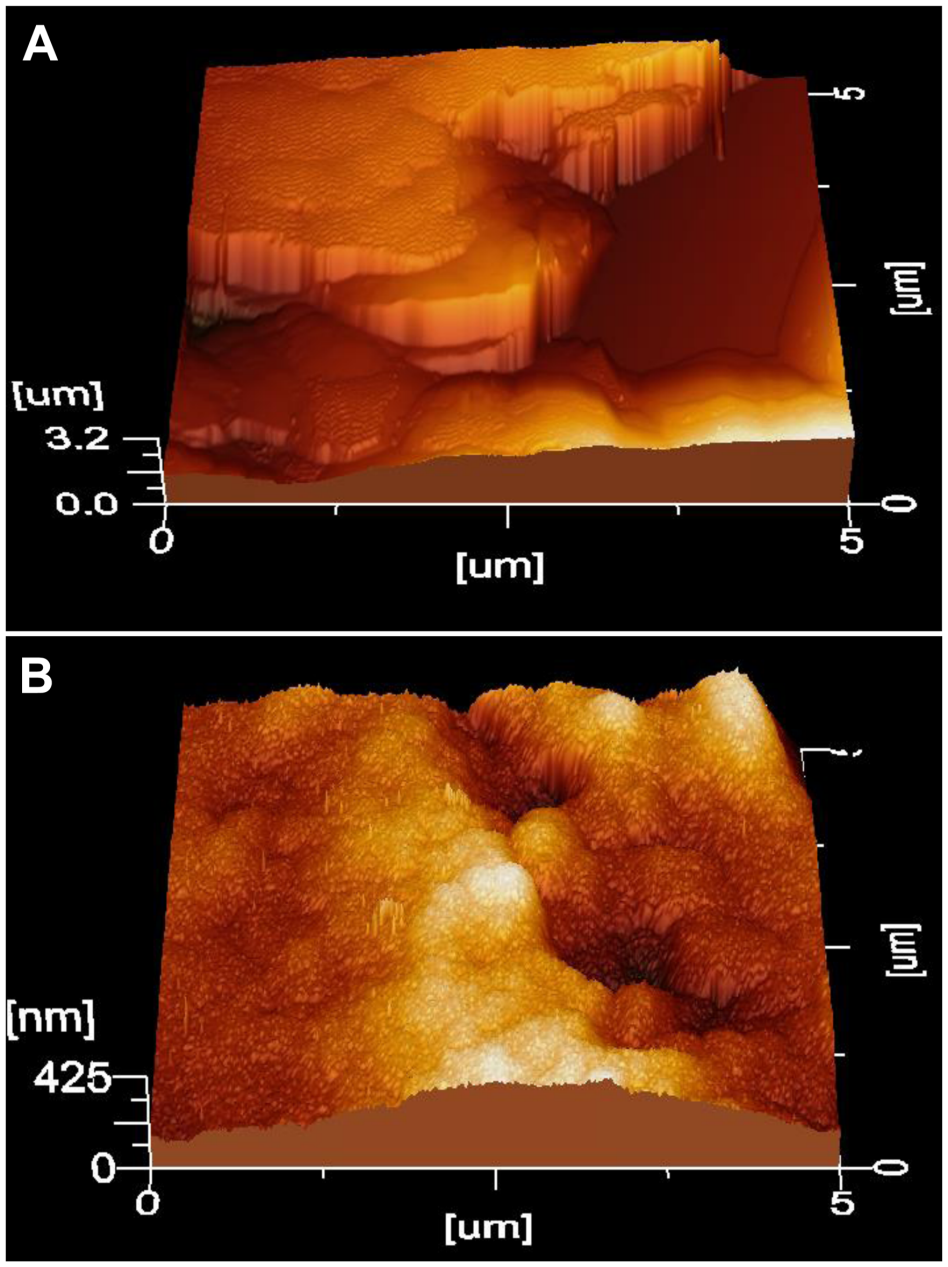
Atomic force microscope phase image in tapping mode of the mineralized tissues filling the rat calvaria defect treated with gelatin matrix containing *hr*CEMP1. (A) Crystals are organized in palisades-like structures. (B) Needle-like crystals developed *in vivo* by *hr*CEMP1 and their features resemble bone.

In order to determine the nature of the mineralized material induced by hrCEMP1 into the calvaria defects, the *in vivo* newly formed bone-like tissue was analyzed by energy dispersive X-ray microanalysis. The inorganic composition as analyzed by x-ray microanalysis revealed a Ca/P ratio of 1.33 (octacalcium phosphate according to ICDD file: PDF#26-1056). The samples analyzed at the nanometer resolution by AFM revealed three-dimensional morphological disposition of the mineralized-like tissue that depicted long ordered three dimensional prisms-like crystals 10 nm in width. The palisade-like structure was ordered in lamellar aggregates resembling bone (Fig. 5A). Other feature of the tissue induced by *hr*CEMP1 into the calvaria defect showed tiny organized needle-shaped crystals in valleys and mineral promontories (Fig. 5B).

### Histomorphometry and µCT

**Figure 6:**
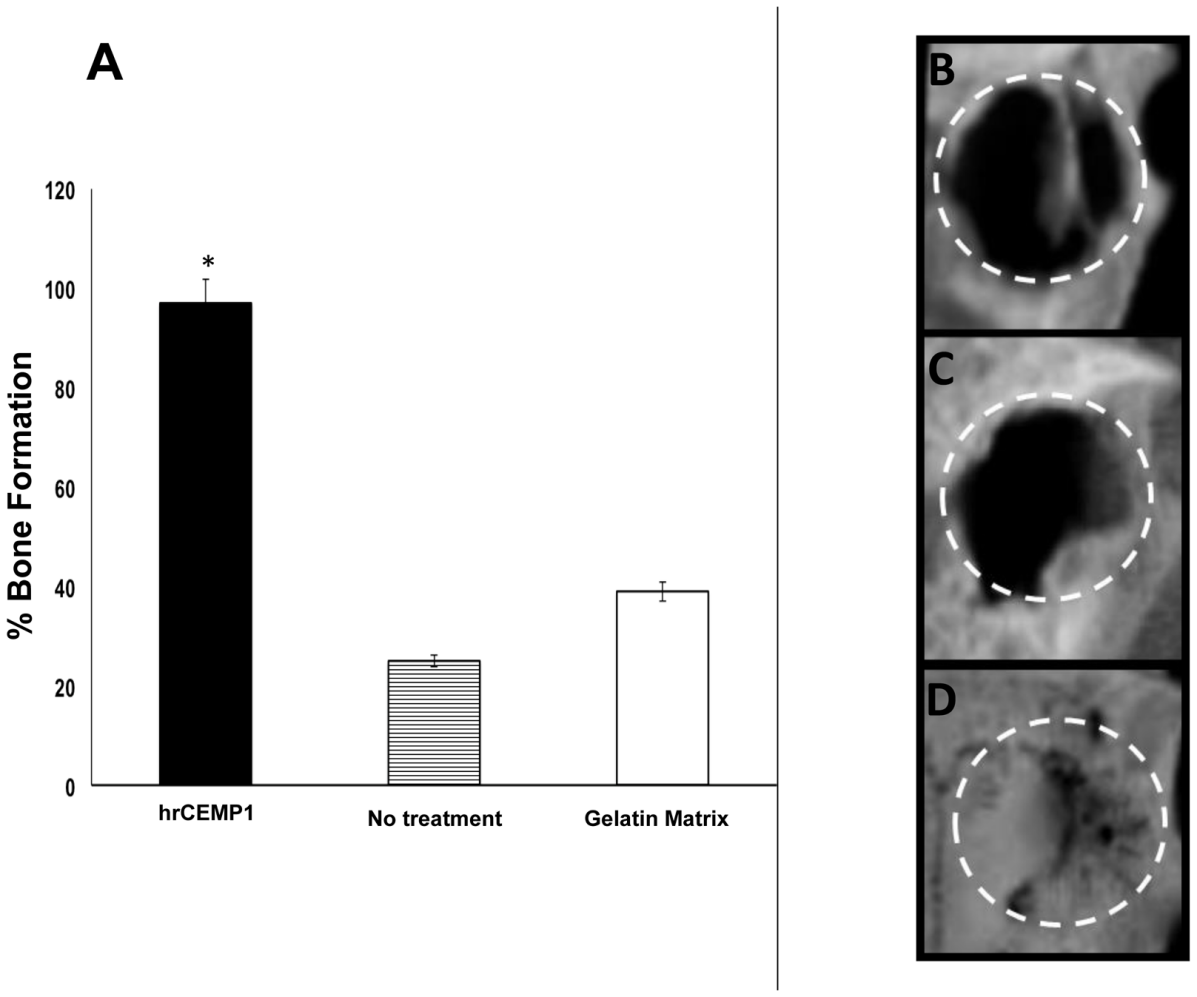
Histomorphometric quantitation of the bone matrix area within the rat calvaria critical-size defect. As can be seen in the graph, *hr*CEMP1 promoted regeneration of the rat calvarial defects up to 97% (A). This can also be seen in the three dimensional µCT of the not treated defect (B), control gelatin matrix scaffold (C) and experimental gelatin matrix scaffold containing *hr*CEMP1 (D) after 16 weeks post-surgery. Image reconstructions were performed at a resolution of 18 µm. The circles indicate the edges of the cranial defect.

In order to quantitatively evaluate bone formation, histomorphometric analysis was performed using H&E stained sections. Samples treated with gelatin matrix scaffolds containing *hr*CEMP1 demonstrated a 97% bridging new bone formation statistically significant as compared to the gelatin only matrix scaffolds and the cranial defect not treated (Fig. 6A). Osteoblasts counts were similar in the rat calvaria host bone (18.3±2.2) and newly formed bone in experimental specimens (26.4±3.6). Control specimens, blank defect and gelatin matrix alone, were filled with fibrous tissue. Three-dimensional microcomputed tomography (µCT) images were also examined to determine the microarchitecture and distribution of the newly formed mineralized tissue. As can be clearly seen, the not treated defect (Fig. 6B) and the gelatin only matrix scaffolds (Fig. 6C) defect, remained largely opened with some minimal mineralized regions characterized by non-uniform low-density shadows at the center of the defect or on regions confined mostly to the defect edges.(Figures 6B and C). In contrast, defects filled with scaffolds containing *hr*CEMP1 displayed almost completely closure with mineralized tissue all throughout the defect showing high homogeneity and high density (Fig. 6D). Although there was some small regions where no bone formation was evident, the edges of the regenerated calvarial defect appears continuous with the surrounding bone. The distribution of mineralization observed with this analysis was consistent with histological examinations.

## Discussion

In the course of several years, we have isolated, purified and characterized a cementum-specific protein known as CEMP1 [16]–[19]. We have demonstrated that CEMP1 has the ability to promote cell differentiation and mineralization, even in cells not destined to produce a mineralized extracellular matrix nodule formation *in vitro* [18]. In the present study we wanted to further determine CEMP1’s biological activities to promote bone formation. Using a well-established model for cranial bone repair, we show that application of a gelatin matrix scaffold containing *hr*CEMP1 in a rat calvaria critical-size defect, promoted bone growth and repair of the defect.

Mineralized tissue healing requires the presence of regenerative cells mineralization promoting proteins such as growth factors, and a conductive local microenvironment where the healing takes place. In the critical-size calvaria defect, osteoblasts or stem cells appear to be induced by CEMP1, acting like a growth factor [18], contained in a gelatin matrix scaffold providing the right microenvironment. The role of CEMP1 as inducer of bone formation is further demonstrated by the inability of the defect to close itself or the gel matrix alone to completely repair the defect.

We show that the CEMP1 induces the nucleation of octacalcium phosphate (OCP) which is a proposed precursor of bone and tooth hydroxyapatite crystals [28], [29]. In fact, OCP has been shown to be somehow effective for bone regeneration of various defects by increasing the osteoblastic cell lineage thus enhancing bone formation [30], [31]. Our evidence showing that CEMP1 nucleates OCP crystals indicates that CEMP1 enhances cell differentiation toward an osteoblastic phenotype and this particular biological characteristic of CEMP1 offers advantages over the use of synthetic OCP to induce bone formation *in vivo* [32]–[35]. Since we demonstrated that *hr*CEMP1 is able to initiate the deposit of octacalcium phosphate, a hydroxyapatite precursor, then, it is conceivable that this phenomenon improves cell/substrate adhesion, since calcium activates osteoblast proliferation and differentiation [36]. Since OCP has received intensive interest as a bone substitute material, little is known about the microstructure of OCP crystals. In our studies *hr*CEMP induces the formation of ordered and random crystal structures. Therefore the OCP microstructure could be a determinant of osteinductive characteristics. However, bone formation by OCP could be stimulated during initial period conversion before the structural maturation into hydroxyapatite [28], [30]–[32]. New bone formation generally seemed to be initiated from the surface of ordered OCP crystals. However, OCP crystals, ordered and random tend to hydrolize into hydroxyapatite and conversion advances in similar velocity and bring out elevated osteogenic capacity, therefore both morphological crystal structures may have an equal potential to induce bone formation [37].

The biomaterials used for bone grafts should provide three dimensional support for cell migration, proliferation, differentiation, and thereby act as a scaffold for new bone formation in defective areas and it must show compatibility and affinity to osteogenic bone matrix proteins [38], [39]. An absorbable collagen sponge has commonly been used as a carrier, has demonstrated suitable kinetics *in vivo* and it is absorbed in 6 weeks [40]–[43]. *In vivo*, gelatin matrix induces the formation of fibrin-rich matrices and therefore it could act as a natural retardant to prolong *hr*CEMP release from the gelatin matrix. Unlike collagen, gelatin does not express any antigenicity in physiological conditions [44]. Gelatin in the form of a highly porous sponge has a long history of use as hemostatic agent [45]–[46], plasma expander [47], and in bone repair [48]. Gelatin matrix scaffolds have been shown to be supportive of chondrogenic matrix production *in vitro* [49], to support osteoblast activities and to allow cell proliferation and cell migration into the sponge porosities. BMP2-incorporated in three-dimensional porous structures of gelatin, has been used for bone regeneration in the clinic [50] and it is effective for osteinduction *in vivo* and *in vitro* [51], [52]. Previously we have determined the optimal concentration of *hr*CEMP1 (2.5 µg) to promote cell proliferation and cell differentiation of human periodontal ligament cells toward a osteoblast-like cells and consequently to produce bone-like tissue *in* *vitro* [19]. Furthermore based on the kinetics of *hr*CEMP1 *in vitro*, previously we determined that 25 µg of *hr*CEMP embedded into the gelatin matrix had an 87% protein constant release after 24 h. Therefore, the remnant protein concentration of ∼ 2.5–3.0 µg into the gelatin matrix seemed to be maintaining its biological activity *in vivo*. Therefore we can infer a long-term effect of *hr*CEMP1.

In the present study gelatin matrix scaffolds containing *hr*CEMP1 enhanced bone regeneration within a cranial critical-size defect compared to that of gelatin matrix scaffold alone and defects left empty during the 16 weeks experimental period. The sham surgery control group produced fibrous connective tissue in most cases rarely bone formation was observed in areas close to the defect margins. Furthermore, gelatin matrix scaffolds containing *hr*CEMP1 displayed a significant increase in both osteoid and mineralized tissue area, when compared to the other two conditions. It has been shown that the potential of recombinant protein technology is crucially dependent upon the characteristics of the carrier [53], [54]. Bone formation with scaffolds containing *hr*CEMP1 was qualitatively and quantitatively superior to that that obtained with gelatin matrix scaffolds and defects left empty.

The bone regeneration results obtained in these studies strongly indicate that *hr*CEMP1 was biologically active and its delivery was sustained. Other studies have shown bone regeneration in short periods of time [55], [56], however, a long-term activity of *hr*CEMP1 is a clear outcome of these studies. Our results clearly support the role for CEMP1 in promoting mineralization and osteogenesis *in vivo*, however, the mechanism is not completely understood. CEMP1 may act as chemoattractant to recruit precursor mesenchymal cells into the defect, and concomitantly acts a differentiation factor for cells to drive bone formation [57]. In an animal dog model for dental pulp necrosis, CEMP1 was shown to recruits mesenchymal stem cells from the periodontal ligament, and promoted proliferation and mineralization of these cells. *In vitro*, *hr*CEMP1 has been shown to promote proliferation and migration of periodontal ligament cells with the migration front comprised of STRO-1-positive cells. [58]. The role of CEMP1 as a chemoattractant and as a promoter of mineralization is further supported by the findings that mineralization was reduced upon blocking CEMP1 function *in vitro*. In cementoblastoma-derived cells, blocking CEMP1 activity decreased ALP activity, BSP and OPN expression without altering cell proliferation [59]. To date, molecules responsible for recruiting mesenchymal cells and inducing their differentiation into cementoblasts have not been identified: these studies suggest that CEMP1 could be one of the molecules.

The histomorphometric analysis revealed complete or almost complete defect closure after 16 weeks for sites receiving *hr*CEMP1. These sites demonstrated re-established cortical plates to the original contour of the calvaria without aberrant reactions such as bone overgrowth. In fact, experiments followed after 1 year show that *hr*CEMP1 does not promote bone overgrowth (Fig. S3). The rat calvaria, critical-size, through-through osteotomy defect appears to be the preferred model to screen candidate osteoconductive and osteoinductive technologies [26]. According to the results of this study, CEMP1 is able to induce the formation of bone-like tissue and *hr*CEMP1 possesses the expected biological activity in promoting bone growth and bone viability demonstrated by osteoblasts facing the osteoid area and by the count of osteocyte-occupied bone lacunae. This is not surprising, since previously CEMP1 has shown to induce phenotypic changes in non-osteogenic cells toward bone-like cells [18]. Therefore, CEMP1 might be a key component that coordinates precisely in temporal and spatial relationships in order to reconstruct the architecture and function of bone and the periodontal tissues previously affected by periodontal disease. In fact, CEMP1 causes progenitor cell differentiation into cementoblast [27], [59] and periodontal fibroblast proliferation and differentiation [19]. In this study, we demonstrate that this function is not only for periodontal regeneration but it can also function for cranial bone repair/regeneration. Therefore, CEMP1 represents an ideal molecule with potential to promote and enhance the regeneration of bone tissues in general, independent of their anatomical location.

The results reported in this study clearly provide strong evidences that *hr*CEMP1 plays a key role during the mineralization process by nucleating octacalcium phosphate crystals and promoting bone regeneration in critical-size defects in rat calvaria. The findings of the present study confirm that *hr*CEMP1 has osteoinductive potential and enhances the physiologic formation and maturation of new bone-like tissue and this novel protein offers new therapeutic venues for the regeneration of bone and other mineralized tissues.

## Supporting Information

Figure S1 Scanning electron microscope image showing a representative microsphere with a diameter of 334 µm (A). Microsphere representing crystals emerging from a mineralized nucleus with an average length of 44 µm (B). Octacalcium phosphate crystals showed a broadness average of 3.64 µm (C). The planar surface of the OCP crystals show a thickness average of 0.89 µm (D)
(PPTX)

Figure S2 Photomicrograph showing the characteristics of normal bone with a blood vessel and osteocytes lacunae (A). Regenerated rat calvaria bone by *hr*CEMP1 shows a well-structured osteon (B). A larger view of normal rat calvaria bone shows bone marrow spaces and osteocytes lacunae (C). Human recombinant CEMP1 induced the regeneration of the rat calvaria bone and shows blood vessels and bone marrow spaces. Photomicrographs A and B were taken at 400x, C and D were taken at 200x.
(PPTX)

Figure S3 Photomicrographs show the characteristics of normal bone rat calvaria (A), rat calvaria critical-sized defect filled with gelatin matrix (B). Rat calvaria ritical-size defect treated with *hr*CEMP1 and evaluated after a year. Bone shows normal histological and anatomical characteristics. Importantly, note that there is not bone overgrowth. Arrows indicate the limits of the defect.
(PPTX)
