# Supplementary figures and images for: Bone Regeneration in Rat Cranium Critical-Size Defects Induced by Cementum Protein 1 (CEMP1)
Source: PLoS One. 2013 Nov 12;8(11):e78807. doi: 10.1371/journal.pone.0078807 (PMC3827101; doi:10.1371/journal.pone.0078807)

## Slide 1
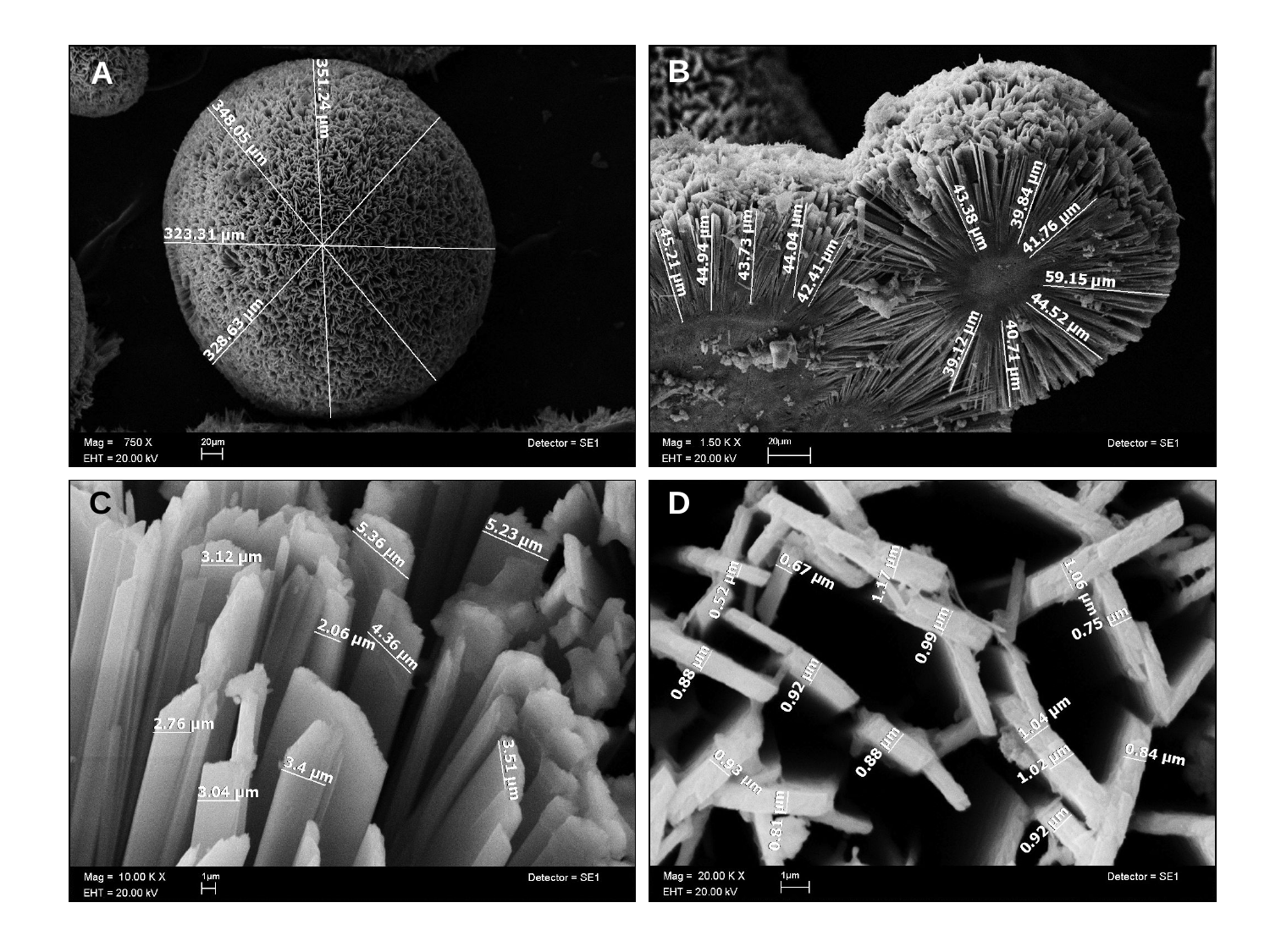

B
A
C
D

Supplement: Figure S1 — Scanning electron microscope image showing a representative microsphere with a diameter of 334 µm (A). Microsphere representing crystals emerging from a mineralized nucleus with an average length of 44 µm (B). Octacalcium phosphate crystals showed a broadness average of 3.64 µm (C). The planar surface of the OCP crystals show a thickness average of 0.89 µm (D) (PPTX) [file pone.0078807.s001.pptx]

## Slide 1
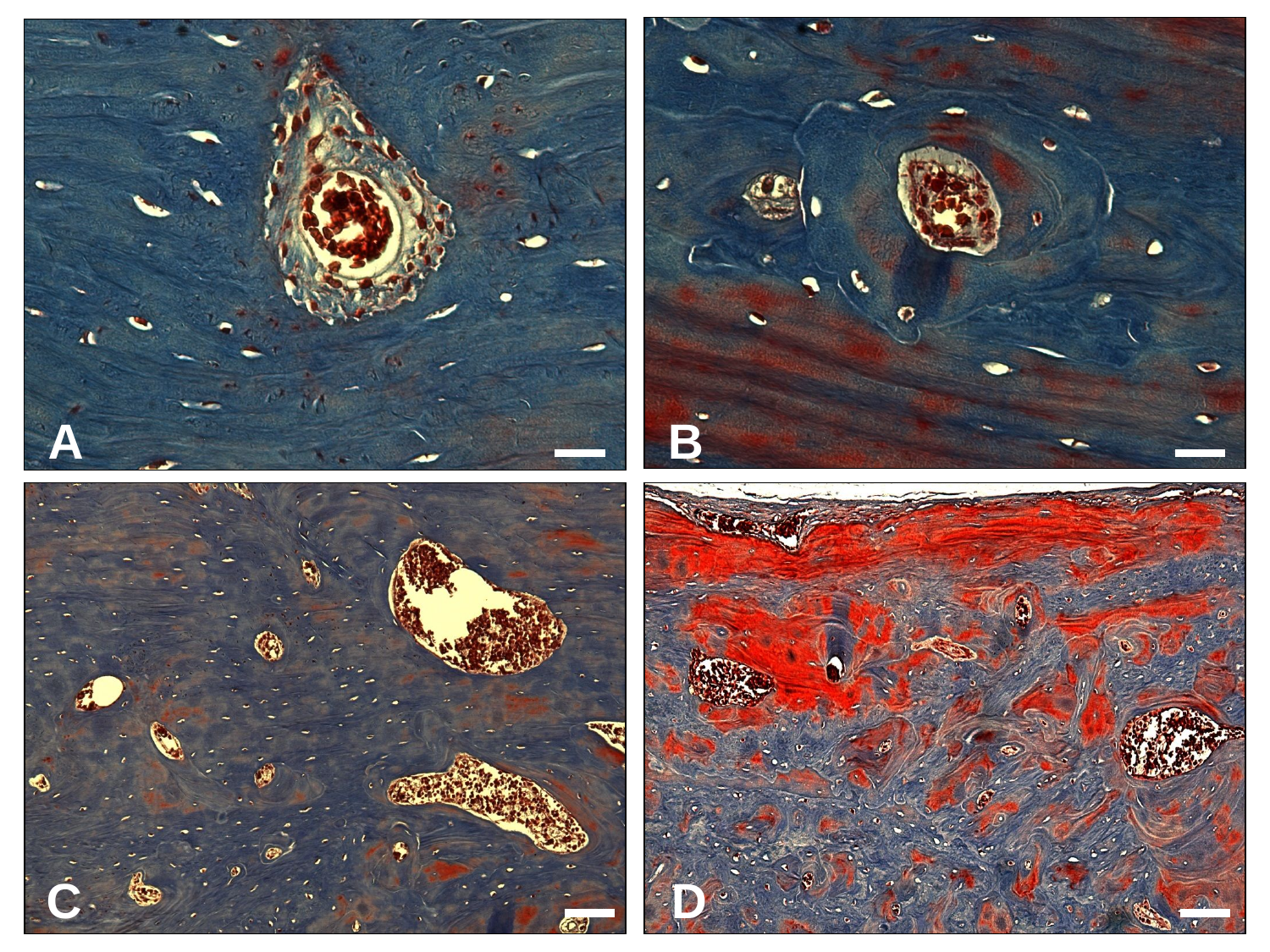

A
B
C
D

Supplement: Figure S2 — Photomicrograph showing the characteristics of normal bone with a blood vessel and osteocytes lacunae (A). Regenerated rat calvaria bone by hrCEMP1 shows a well-structured osteon (B). A larger view of normal rat calvaria bone shows bone marrow spaces and osteocytes lacunae (C). Human recombinant CEMP1 induced the regeneration of the rat calvaria bone and shows blood vessels and bone marrow spaces. Photomicrographs A and B were taken at 400x, C and D were taken at 200x. (PPTX) [file pone.0078807.s002.pptx]

## Slide 1
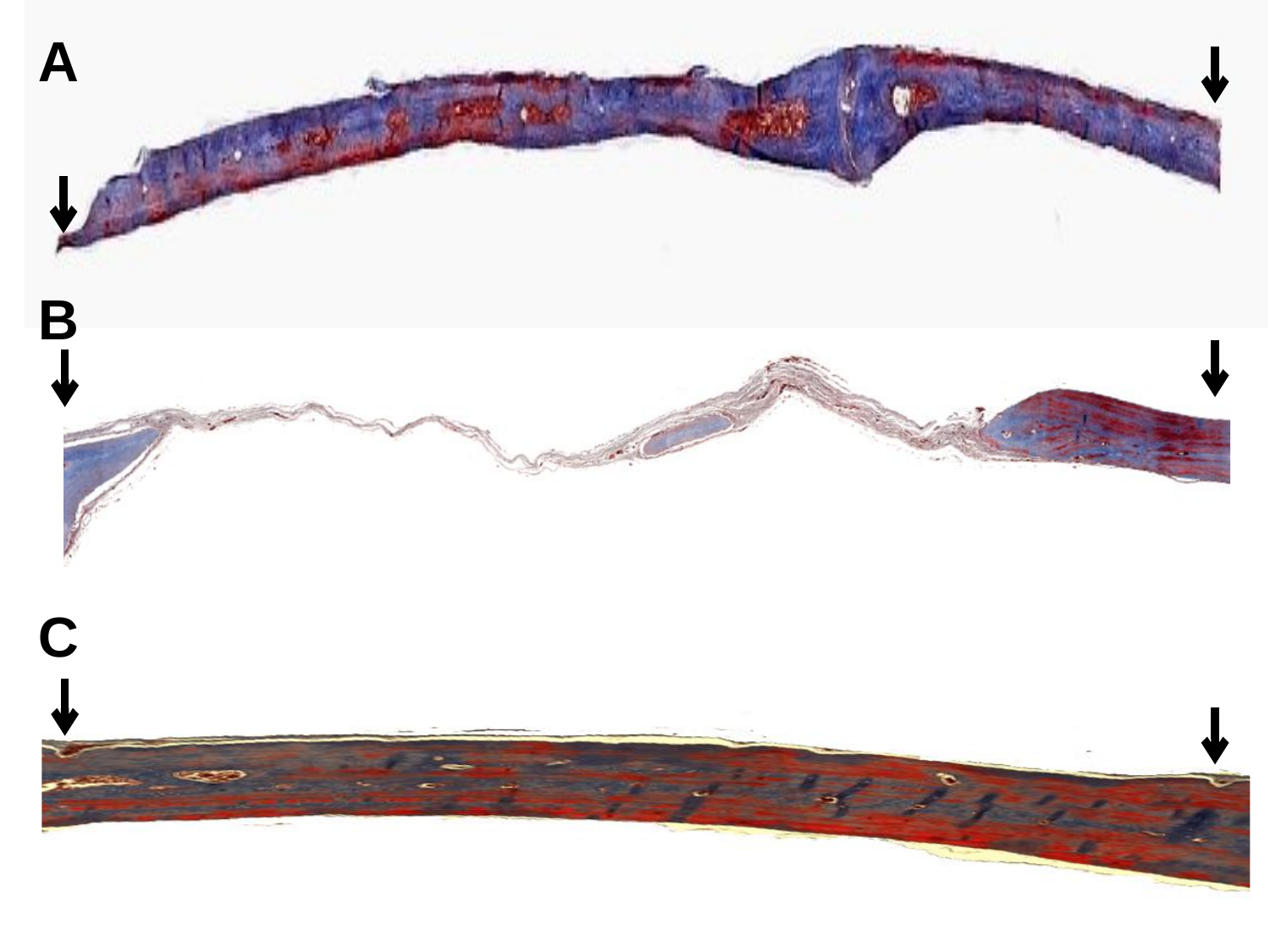

A
B
C

Supplement: Figure S3 — Photomicrographs show the characteristics of normal bone rat calvaria (A), rat calvaria critical-sized defect filled with gelatin matrix (B). Rat calvaria ritical-size defect treated with hrCEMP1 and evaluated after a year. Bone shows normal histological and anatomical characteristics. Importantly, note that there is not bone overgrowth. Arrows indicate the limits of the defect. (PPTX) [file pone.0078807.s003.pptx]
